# The rise of antifeminist perspectives among future educators: a growing concern?

**DOI:** 10.3389/fpsyg.2025.1585199

**Published:** 2025-06-13

**Authors:** Nahia Idoiaga-Mondragon, Idoia Legorburu Fernandez, Maitane Picaza Gorrotxategi, Israel Alonso Saez

**Affiliations:** ^1^Department of Evolutionary and Educational Psychology, University of the Basque Country UPV/EHU, Leioa, Spain; ^2^Department of Didactics and School Organization, University of the Basque Country UPV/EHU, Bilbao, Spain

**Keywords:** feminism, antifeminism, university students, educators, gender perspective

## Abstract

**Introduction:**

Antifeminist discourses have historically emerged in response to feminist advancements. In Spain, the past decade has seen significant feminist progress, yet antifeminist rhetoric has simultaneously gained traction, particularly among younger populations. This study examines the antifeminist narratives present among university students in education programs, a critical demographic as future educators play a key role in shaping social attitudes.

**Methods:**

A free association exercise was conducted with 252 students enrolled in education programs. The collected textual data were analyzed using Iramuteq software, applying lexical analysis techniques to identify dominant antifeminist discourses.

**Results:**

The findings indicate that the most prominent antifeminist narratives among participants center on feminist principles, salary aspirations, perceived disparities in rights, household chores, and the alleged provocative behavior of women. Notably, frequently cited statements include “*Feminism does not seek equality”* and “*They expect to be paid the same”*, reflecting common misconceptions about feminism. Despite expressing support for gender equality, participants were less likely to self-identify as feminists.

**Discussion:**

These findings highlight the contradictions within young adults' perceptions of feminism and gender equality. The persistence of antifeminist narratives within future educators underscores the need to address these discourses in academic settings. Integrating feminist pedagogies into teacher training programs is essential to fostering a more equitable and informed educational environment.

**Conclusion:**

This study underscores the necessity of actively confronting antifeminist rhetoric within educational institutions to ensure that future educators contribute to gender equality rather than perpetuate misconceptions.

## 1 Introduction

### 1.1 Feminism: waves and political strands

Feminism is one of the most significant social movements in recent industrialized societies, sparking societal debate (Delmar, 2018; Loke et al., 2017). This social and political movement is rooted in the pursuit of equal rights for women and men, alongside the eradication of gender-based discrimination (Arat, 2015; Bhattacharjya et al., 2013; Parisi, 2002; Zembat, 2017). It emerged in response to the systematic inequalities and oppressions that women have faced throughout history (Connelly et al., 2000; Ferree and Mueller, 2004). Over time, feminism has evolved through different waves and approaches, yet its fundamental objectives remain consistent: advocating for gender equality, women's empowerment, and, consequently, global justice (Cornwall and Rivas, 2015).

While formal feminism emerged in the late eighteenth century, it is important to recognize that throughout history, many women questioned the societal roles imposed upon them and challenged prevailing conventions. However, these women are not typically categorized within any specific feminist wave, as their contributions were often individual and lacked the collective awareness of fighting for a shared cause, as seen later with the first-wave feminists (Rowbotham, 2013).

It is uncommon to speak of a “wave” of feminism in the modern sense during the eighteenth century. However, that period witnessed significant events and figures that helped lay the foundations for the later development of the feminist movement, especially during the Enlightenment, when some thinkers began to question traditional notions of women's inferiority (Lerner, 1993). The contributions of figures such as Olympe de Gouges or Mary Wollstonecraft were notable (Crenn, 2019). Nevertheless, the powers that be reacted very negatively to this first enlightened feminist debate, leading to the exclusion of women from political rights (Spencer, 2012).

While Anglo-Saxon feminist theories have largely shaped Western academic discourse, it is essential to acknowledge other feminist traditions that have significantly contributed to the struggle for gender equality. In the Iberian Peninsula, for instance, the Enlightenment period witnessed the emergence of feminist voices that questioned the subordination of women and advocated for their access to education, intellectual development, and civic participation (Smith, 2006). These early contributions, often overlooked in mainstream narratives, remind us of the importance of situating feminist movements within diverse historical and geographical contexts (Bermúdez and Johnson, 2018).

The first wave of feminism, often referred to as suffragism, emerged in the mid-nineteenth century in the United States and the United Kingdom. During this phase, feminism transitioned from a purely intellectual endeavor to a mobilized social movement (Sanders, 2004). However, this wave was not monolithic (Rome et al., 2019). Internal debates emerged regarding strategies (constitutionalism vs. militancy), the inclusion of working-class women and racial minorities, and differing priorities between political and social rights (Mayhall, 2000). Suffragism was primarily led by white bourgeois women. By the late nineteenth and early twentieth centuries, women's suffrage became a reality (DuBois, 1998). However, the notion of granting legal and civil rights to women, including the right to vote, faced resistance from various fronts or groups, such as anti-suffragists, social conservatism, religious opposition, and antifeminist movements in the press (Bush, 2007; Steuter, 1992). In Spain, women's suffrage was granted in 1931, during the Second Spanish Republic. However, the Franco dictatorship that followed the Civil War vehemently opposed it, and Spanish women did not reclaim this right until the general elections of June 1977, following the demise of the Franco regime (Aguado, 2012).

The second wave of feminism, occurring roughly in the 1960s and 1970s, marked an expansion and evolution of the feminist movement. This phase was characterized by addressing a broader range of issues related to gender equality and women's liberation, including workplace equality, reproductive rights, anti-discrimination efforts, challenging traditional gender roles, and raising awareness of gender-based violence (Thornham, 2004). During this period, feminism diversified into multiple strands, including radical feminism, which focused on structural patriarchy; liberal feminism, advocating legal reforms; socialist feminism, addressing class and gender intersections; and ecofeminism, linking environmental and gender struggles (della Porta and Bonu Rosenkranz, 2025). These currents often coexisted in tension, reflecting the movement's heterogeneity (Mann and Huffman, 2005). Like the first wave, the second wave encountered resistance and opposition from various sectors of society (Whelehan, 1995). During this period, conservative feminism continued to gain traction, giving rise to pro-life groups critical of reproductive rights movements, resistance to changes in gender roles, and strong criticism from conservative and religious groups against sexual liberation, among other challenges (Burdick, 2023; Foxworth, 2018). The second wave of feminism in Spain had a significant impact in the 1970s and 1980s, coinciding with a period of political and social transformation in the country (Larrondo Ureta, 2020).

The third wave of feminism emerged in the 1990s and extended into the first decades of the twenty-first century (Snyder, 2008). This wave arose in response to perceptions that the struggles and achievements of second-wave feminism did not adequately address the diverse experiences and challenges faced by women in various contexts (Heywood and Drake, 1997). These challenges included intersectionality, sexuality and sexual empowerment, body image, popular culture, reproductive justice, gender-based violence, sexual harassment, leadership and political participation, and the use of social networks and the internet (Gillis et al., 2004; Snyder, 2008, 2010; Tiwari, 2023). The third wave also coincided with the rise of post-feminism, which re-signified feminist ideas through neoliberal, individualistic lenses, often focusing on personal choice and empowerment while sidelining collective and structural critiques (Gill, 2007). This overlap generated tensions and debates about the future directions of feminism (Snyder, 2008).

From this third wave, activists and academics have identified the emergence of a fourth wave of feminism in the second decade of the twenty-first century. This wave is characterized by its theoretical foundations in combating violence against women (Barriga, 2020; Munro, 2013; Rivers, 2017). Moreover, it is strongly linked to online activism and movements such as #MeToo while also drawing from intersectionality, diversity, and ecofeminism (Tiwari, 2023).

### 1.2 Feminism in Spain: achievements and controversies

In Spain, feminist movements have experienced significant empowerment in the past decade, notably highlighted by the massive demonstrations of the feminist strikes on March 8, 2018, and 2019 (Idoiaga Mondragon et al., 2022). Additionally, large-scale protests and demonstrations ensued in response to the lenient sentencing of the perpetrators in the “La Manada” gang rape case (Idoiaga Mondragon et al., 2020). Moreover, many advancements championed and realized by the Ministry of Equality during the 14th legislature, such as the “Only yes, is yes” law or the “Trans law,” have further bolstered the feminist movement, garnering support even within feminist circles (Sanz, 2023).

The “Only Yes is Yes” law (Organic Law on the Comprehensive Guarantee of Sexual Freedom), approved in 2022, redefined sexual consent in Spain, establishing that consent must be affirmative, explicit, and cannot be assumed in ambiguous situations. This legislative change sought to address gaps in previous legal frameworks that required evidence of violence or intimidation to qualify an act as sexual assault. However, the law's implementation led to significant controversy due to certain technical aspects of the penal code reform, which inadvertently resulted in sentence reductions for some convicted offenders. This sparked a wave of criticism from conservative political sectors, mainstream media, and even within parts of the judiciary (Asensi-Rodríguez and Martínez-Rolán, 2024). The ensuing backlash fueled antifeminist narratives, framing the law as an example of ideological excess and incompetence, thereby amplifying social polarization regarding gender equality policies (Rivas Venegas, 2021).

Similarly, the approval of the “Trans Law” (Law for the Real and Effective Equality of Trans People and Guarantee of LGTBI Rights) in 2023, which facilitates gender self-identification, has also been a source of intense public and political debate. Besides opposition from conservative sectors, this law has provoked internal tensions within feminist movements, particularly between trans-inclusive perspectives and trans-exclusionary radical feminist positions (Platero, 2023). These debates have further polarized public discourse on gender and feminism in Spain, contributing to a fertile ground for antifeminist narratives (Pérez, 2024).

However, these advancements have also sparked antifeminist movements, primarily rooted in the most conservative political parties, which have repeatedly opposed the feminist movement (Bernardez-Rodal et al., 2022; Cabezas, 2022). Beyond the political sphere, certain sectors of the judiciary have demonstrated resistance to feminist legislative changes, especially evident in the judicial application of the “Only Yes is Yes” law, where controversial rulings reignited debates about institutional sexism and the role of the judiciary in gender equality policies (Rincón, 2022). Additionally, mainstream media have played a key role in amplifying antifeminist narratives, often portraying feminist policies as ideological impositions disconnected from societal needs, thereby fostering the stigmatization of feminist movements in public opinion (Valdés, 2023).

### 1.3 Contemporary antifeminist discourses

However, attributing these antifeminist movements solely to the extreme right would be inaccurate. It is interesting to note the research conducted on general populations, especially young people, who have historically been closely associated with the feminist movement. In particular, several studies from the last decade indicate a lack of engagement with feminism among young people in democratic societies (Elizalde and Álvarez, 2021; Gómez-Ramírez and Reyes, 2008). Indeed, some scholars argue that many young individuals may not identify as feminists and even dissociate themselves from feminism (McRobbie, 2004), partly due to the stigma attached to feminist movements in certain sectors of society (Aronson, 2003). Feminism has been criticized and misrepresented, with feminists being portrayed as reverse sexists, unfeminine “feminazis” (Anderson et al., 2009; McCabe, 2005), man-haters (Aronson, 2003; Houvouras and Carter, 2008), or as advocating for female superiority over men (Ramsey et al., 2007; Reid et al., 2018; Toller et al., 2004).

Similarly, findings from recent surveys shed light on prevailing attitudes toward feminism in Spain. According to a survey conducted by the state Sociological Research Center in January 2024, 44.1% of men and 32.5% of women strongly or somewhat agree that the progress made in promoting women's equality has led to discrimination against men (CIS, 2024). Similarly, a study focusing on university students in the same region revealed that while the majority of students acknowledge the positive impact of the feminist movement and its work, a significant portion—one-quarter of them—perceive feminism as a radical social movement that is distant from their own beliefs (Fernandez et al., 2023).

In the current socio-political context, emerging generations are growing amidst persistent tensions surrounding feminism, antifeminism, and post-feminism. Consequently, openly proclaiming oneself as a feminist in public entails a multifaceted process of intellectual and emotional negotiation (Adams et al., 2007). Analyzing the perceptions of feminism or antifeminism among younger generations and exploring their identification with feminist ideals can provide valuable insights for developing educational strategies that foster equality values among young individuals (Jackson, 2018; Fernández Rotaetxe et al., 2021).

Despite efforts by various stakeholders in the educational sphere, such as teachers, gender educators, and high school gender officers, concerns have been raised regarding the barriers and antifeminist discourses emerging from the student body in recent years (Luque, 2021; Sádaba et al., 2023). These newly emerged discursive ideas often refute gender inequality, asserting that feminism exaggerates or fabricates it and suggesting that we live in a just society where there is no longer a need to advocate for women's rights (Elder et al., 2021). Moreover, they reject the term “feminism,” viewing it as a movement exclusively for women that fails to acknowledge the challenges faced by men (Ging and Siapera, 2019).

However, despite some institutions initiating specific campaigns to address these issues (see Intered, 2024), there remains a lack of studies that have thoroughly analyzed the prevailing context, with even fewer focusing on soliciting the perspectives of young people regarding the antifeminist ideas they encounter in their daily lives. Within this framework, our research aims to examine antifeminist ideologies among education students.

### 1.4 Relevance of the study and objectives

The relevance of this study lies in its dual contribution to both higher education and institutions dedicated to feminist training in diverse fields, including secondary education, high schools, and leisure or extracurricular activities. By focusing on the opinions, knowledge, and social representations of university students in education degrees, this research addresses a critical dimension of teacher training strategies. These students, as future educators, will soon play a pivotal role in shaping the educational experiences of children and adolescents. Understanding their perceptions regarding gender equality and antifeminist discourses is, therefore, of paramount importance for the development of effective educational interventions.

The potential benefits of this study include providing an empirical foundation for the design of training strategies aimed at promoting equality and feminist values, both within university curricula and in broader educational or recreational contexts. The findings will offer a comprehensive overview of the current beliefs and representations held by young students regarding antifeminist ideologies, serving as a diagnostic tool to inform future educational policies and initiatives.

The primary objective of this research is to identify the prevailing antifeminist ideologies among education students and to assess the extent to which these discourses are endorsed or rejected. By analyzing how these future educators perceive and position themselves in relation to antifeminist narratives, the study seeks to generate valuable insights into the challenges and opportunities for fostering gender equality within teacher training programs.

In order to achieve this overarching aim, the study will address the following specific objectives:

- Specific Objective 1: To identify the antifeminist discourses prevalent among young people.
- Specific Objective 2: To analyze the degree of identification with these discourses among young people themselves, their friends, and youth in general.
- Specific Objective 3: To assess whether the representation of antifeminist discourses varies according to participants' self-identification as feminists or the importance they attach to gender equality.
- Specific Objective 4: To investigate whether the representation of antifeminist discourses differs based on participants' age or gender.

Given the exploratory nature of this research, no *a priori* hypotheses have been established.

## 2 Methodology

To achieve the objectives proposed for this project, qualitative and quantitative data have been collected through online questionnaires specifically designed for this purpose. These surveys include open-ended and closed-ended questions to gather comprehensive responses from participants.

### 2.1 Sample

The sample comprised 252 education students from the XXXXXX. The mean age of the participants was 21 years (sd = 5.64). Regarding gender distribution, most of the sample identified as women, accounting for 72.6%, while 27.4% identified as men.

Regarding the distribution by degree, the largest proportion of students was pursuing degrees in Primary Education (38.89%), followed by Early Childhood Education (28.17%), and the Master in Teacher Training of Compulsory Secondary Education and Baccalaureate, Vocational Training, and Language Teaching (21.8%). A smaller percentage of students were enrolled in degrees of Social Education (9.13%) and Pedagogy (2.38%). Regarding academic year, the distribution was as follows: 30.15% were first-year students, 28.17% were second-year students, 11.90% were third-year students, 7.94% were fourth-year students, and 21.83% were master's degree students.

When asked to rate their self-categorization as feminists on a scale of 1–5, the participants obtained an average score of 3.96 (sd = 0.97). Participants were also asked to rate their support for gender equality on the same scale. On average, they reported a score of 4.83 (sd = 0.48).

### 2.2 Compliance with ethical standards

The study sample was recruited from the Faculties of Education at the University of the Basque Country. Before data collection, approval was obtained from the University Ethics Committee [Ref.:M10/2023/141]. All participants volunteered to participate in the study and were provided with detailed information about the research procedures. They provided informed consent before participating. Recruitment was conducted using non-probabilistic snowball sampling. A questionnaire was created and disseminated through various channels, including virtual platforms, social networks, and university emails sent out by the researchers.

### 2.3 Instrument

The questionnaires were structured into two parts. Firstly, participants were requested to provide specific socio-demographic information tailored for this study. This included age, gender (with options for Male, Female, or Non-binary), educational field of study, current course or academic year, and self-reported levels of feminist self-identification and support for gender equality. These two aspects were assessed using Likert scale questions, with respondents asked to rate their identification as feminists and their support for gender equality on a scale of 1–5.

Subsequently, a free-association exercise was administered based on the Grid Elaboration Method (GEM) to analyze participants' social representations of antifeminist ideas (Joffe and Elsey, 2014). This methodology, previously employed in studies exploring the shared representations of young people on various feminism-related topics (Fernández Rotaetxe et al., 2021; Fernandez et al., 2023), was chosen for its effectiveness in eliciting spontaneous responses. Specifically, participants were asked to write down the first four antifeminist words or ideas that came to their minds. Each word or idea was then recorded in a separate box, with participants asked to fill in the four empty boxes provided. Following this, participants were prompted to explain their chosen words or ideas precisely, clarifying their meanings in detail. These explanations formed the basis for subsequent analysis. Finally, in addition to providing explanations, participants were asked to rate their personal agreement with each antifeminist idea on a scale of 1–5 (To what degree do you agree with that X idea you just mentioned?) as well as their perceptions of their friends' agreement (To what degree do your friends agree with that X idea you just mentioned?) and agreement of the broader collective with the antifeminist idea (To what degree do young people in general agree with that X idea you just mentioned?).

### 2.4 Analysis

To examine the collection of open-ended responses, we employed the Iramuteq software to conduct a lexical analysis (Marchand and Ratinaud, 2012). Two distinct analyses were conducted using this software. The initial analysis followed the Reinert method, while the second involved a lexical similarity assessment for each identified idea.

The primary analysis for qualitative variables involved the application of the Reinert method (Reinert, 1983, 1990) using the Iramuteq software. This method is widely recognized and utilized in the examination of open-ended questions across various fields (Legorburu et al., 2022; Idoiaga Mondragon et al., 2022; Souza et al., 2018) and has demonstrated effectiveness in addressing reliability and validity concerns in text analysis as evidenced by previous studies (Klein and Licata, 2003).

The Reinert method follows a top-down hierarchical cluster analysis approach, extracting classes and statistical indicators such as typical words and text segments (Idoiaga and Belasko, 2019). Specifically, the Iramuteq software identifies words and text segments with the highest Chi-square values, signifying those that best represent each class or idea frequently mentioned by participants.

Consistent with previous applications of the Reinert method (Camargo and Bousfield, 2009), the raw data were entered into the Iramuteq software. Significant vocabulary items in each class were selected based on three criteria: (1) an expected word value exceeding 3; (2) Chi-square statistical evidence of association with the class (χ^2^ ≥ 3.89, *p* = 0.05, d*f* = 1); and (3) the word predominantly appearing in that class with a frequency of 50% or more. The Iramuteq software also identified text segments associated with each class, ranking them according to their chi-square values. This study gathered text segments with the most significant chi-squares in each class.

Following the identification of these “lexical universes,” they were linked to “passive” variables (independent variables). In this study, the passive variables were gender, academic year, feminist identification, identification with gender equality, personal agreement, close group (friends) agreement, and collective agreement.

Consequently, the analyst derives a series of classes comprising typical words and text segments (quotes) with the highest chi-square values, forming the basis for interpreting the classes as lexical worlds.

The Reinert method yields statistical, transparent, and reproducible data up to the point of interpretation, where the analyst assigns labels. In the final phase, researchers assigned titles to the sets of words and text segments grouped by the software, following a systematic process. Two researchers independently named each class based on associated words and quotes, with a third researcher creating a final label approved by all three researchers.

## 3 Results

**Figure 1:**
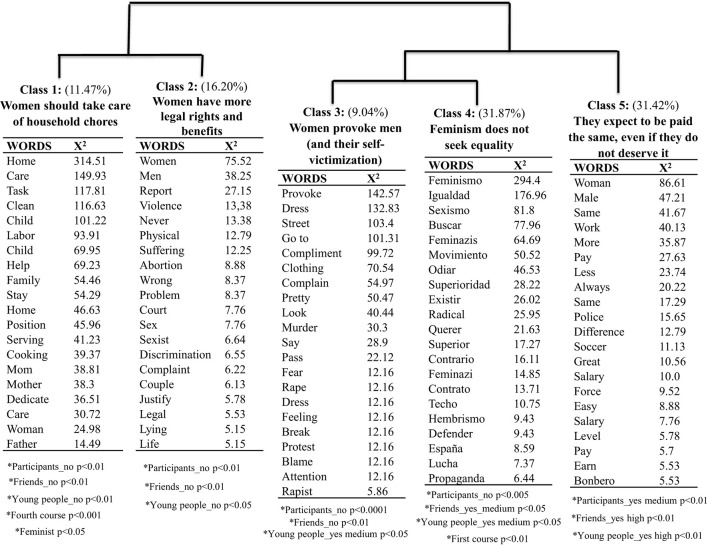
The hierarchical clustering dendrogram showing the most frequent words and those with the greatest association $\chi_{\left(1\right)}^{2}$, *p* < 0.001 extracted by the Reinert method.

The Reinert method, utilizing a descending hierarchical analysis, was employed to identify the primary antifeminist ideas articulated by the participants. Each issue or concept is encapsulated by a collection of characteristic words and text segments referred to as a class. The analysis segmented the corpus into 998 sections, yielding five distinct classes, as illustrated in Figure 1. These classes will be examined individually in the subsequent sections of this article.

### 3.1 Women should take care of household chores

The first idea mentioned by the participants, with a weight of 11.47%, is the belief that women should take care of household chores. However, it should be noted that neither the participants (*p* < 0.01) nor their friends (*p* < 0.01) or young people in general (*p* < 0.05) agree with this statement. Therefore, it can be regarded as an idea prevalent in society but not representative of young people.

The most significant phrases used by the participants to explain this idea were:

- *Women have to be housekeepers and, therefore, take care of the children and do household chores (X*^2^ = *720.50)*.
- *Women have an innate ability to take care of children and do housework, unlike men (X*^2^ = *705.14)*.
- *Women should stay at home doing housework (X*^2^ = *554.91)*.
- *Both housework and childcare should be done by women (X*^2^ = *548.71)*.
- ….

### 3.2 Women have more legal rights and benefits

The second idea mentioned by the participants, with a weight of 16.20%, concerns social and legal rights. The participants assert that women have more social and legal rights than men and that they often take advantage of them. However, the participants themselves (*p* < 0.0001) and their friends (*p* < 0.01) disagree with this statement. Interestingly, young people (*p* < 0.05) moderately agree with this statement (scoring 3 on a scale from 1 to 5).

The most significant phrases used by the participants to explain this idea were:

- *Women today have more rights than men just because they are female, and therefore, in a lawsuit, they will win even if the man is right (X*^2^ = *245.41)*.
- *Men suffer the same amount of violence as women or more (X*^2^ = *206.65)*
- *Antifeminist phrases that people say the most are: “Men and women have the same rights”, “feminism promotes radical feminism”, and “many women file false accusations”. However, these cases are actually very rare and often do not go forward (X*^2^ = *205,65)*.
- *There are a lot of false allegations nowadays. Women can do this with total impunity and harm innocent people (X*^2^ = *204.75)*.
- *With the new law, many men believe that it is almost impossible to talk to women because they will be reported at the slightest opportunity, and there will be serious consequences (X*^2^ = *198.45)*.
- *Women lie and file false reports about gender violence because the law protects them (X*^2^ = *194.65)*.
- *There is no male violence in our society, only violence; the man who abuses a woman does so because he has specific psychological problems that have nothing to do with male chauvinism (X*^2^ = *192.45)*.
- *Women take advantage of the image of weakness that society gives them to make false reports regarding gender violence (X*^2^ = *165.54)*.
- *Men never get custody of their children, and there is, therefore, discrimination against men (X*^2^ = *160.43)*.
- …

### 3.3 Women provoke men (and their self-victimization)

The third idea mentioned by the participants, accounting for 9.04% of the weight, pertains to the notion that women should be mindful of their behavior in public spaces, as they may provoke men and then become offended when men respond. Interestingly, this idea was more frequently mentioned by fourth-grade participants (*p* < 0.001), and those who identified as feminists reported encountering it more often (*p* < 0.05). Despite expressing disagreement with this statement themselves (*p* < 0.005), participants believe that both their friends (*p* < 0.05) and young people in general (*p* < 0.05) moderately agree with it (scoring 3 on a scale of 1–5).

The most significant phrases used by the participants to explain this idea were:

- *Depending on how we are dressed, we are asking for or causing certain inexcusable things to happen to us (X*^2^ = *522.86)*.
- *If a woman in a discotheque dances in a certain way or dresses in a certain way, it means she does it because she wants to provoke (X*^2^ = *429.79)*.
- *If you provoke or go to dangerous places, then it is normal that things happen to you (X*^2^ = *406.32)*.
- *A woman is sexualized by the way she dresses and blamed for it because she is provocative (X*^2^ = *396.57)*.
- *Women love to be complimented and shouted at when we walk down the street (X*^2^ = *304.15)*.
- *I have heard that some rapes are provoked by women because of the way they dress (X*^2^ = *299.65)*.
- …

### 3.4 Feminism does not seek equality

The fourth and most frequently mentioned idea, with a weight of 31.87%, is that feminism does not aim for equality but rather seeks the superiority of women over men. This idea is more commonly mentioned by younger students, that is, those in their first year (*p* < 0.01). Although participants express disagreement with this statement themselves (*p* < 0.005), they believe that both their friends (*p* < 0.05) and young people in general (*p* < 0.05) moderately agree with it (scoring 3 on a scale of 1–5).

The most significant phrases used by the participants to explain this idea were:

- *Today's feminists do not seek equality, many believe that feminism is now a fashion and that what people seek is not equality but radical feminism (X*^2^ = *658.46)*.
- *The most antifeminist idea I have heard is that feminism does not seek equality between men and women but rather the superiority of the female sex (X*^2^ = *643.87)*.
- *There are people who say that neither machismo nor feminism, but rather equality, is sought, and this implies that feminism does not seek equality (X*^2^ = *630.07)*.
- *Feminism seeks the destruction of men, neither machismo nor feminism. I believe in equality (X*^2^ = *617.45)*
- *Current feminism does not seek equality but the superiority of women, generally by diminishing men or making them feel inferior in a vague attempt to propel women toward equality. They are feminazis (X*^2^ = *572.50)*.
- …

### 3.5 They expect to be paid the same, even if they do not deserve it

The fifth idea, comprising 31.42% of the total weight and the second most mentioned by participants, is associated with the realm of work. Participants express the belief that women receive positive discrimination in the workplace, receiving higher pay or equal pay despite not being able to perform all tasks as men do. Additionally, they perceive women as having advantages in certain sectors of work.

This idea is more frequently mentioned by students who consider themselves feminists to a moderate extent (scoring 3 on a scale of 1–5). Moreover, participants themselves express moderate support for this idea (scoring 3 on a scale from 1 to 5). However, they believe that both their friends (*p* < 0.05) and young people in general (*p* < 0.005) strongly support it (scoring 4 or 5 on a scale from 1 to 5).

The most significant phrases used by the participants to explain this idea were:

- *Women who play soccer and get paid more than what they generate doing the exact same job are overpaid (X*^2^ = *295.24)*.
- *The idea that women today are paid less than men for the same job is a lie (X*^2^ = *281.41)*.
- *The entrance examinations for the state security and protection corps or firefighters are easier for women, and technically, they must perform the same job since they are expected to receive the same salary (X*^2^ = *266.04)*.
- *Women should not be paid the same as men when joining the police or fire service because their physical tests are less demanding (X*^2^ = *265.44)*.
- *Women do not deserve the same money as men for exactly the same work and effort because, in sports, you earn what you generate (X*^2^ = *259.37)*.
- *Men generate more revenue, so women cannot be paid the same (X*^2^ = *217.58)*.
- …

### 3.6 Other variables of analysis

First, we would like to re-analyze the distinction between feminist self-categorization and being in favor of equality. When participants were asked to rate their degree of self-identification as feminists on a scale of 1–5, the mean was 3.96 (sd = 0.97). In contrast, when asked about their degree of support for equality between women and men on the same scale, the average response was 4.83 (sd = 0.48). Furthermore, participants were asked to rate their agreement with each of the ideas they mentioned on a scale of 1–5, where 1 represents no agreement, and 5 represents complete agreement. On average, they reported an agreement level of 1.38 (sd = 0.72) with their own ideas. Regarding their perceptions of their friends' agreement with the mentioned ideas, participants reported an average of 2.06 (sd = 0.80). Lastly, when asked about their beliefs regarding the agreement of young people in general with the mentioned ideas, participants reported an average score of 3.36 (sd = 0.80).

## 4 Discussion

This study aimed to identify the prevailing antifeminist ideologies among young education students and assess their level of endorsement or rejection of these ideologies. The findings, derived from the perspectives of these young individuals, identified five primary antifeminist ideas: (1) Feminism does not pursue equality; (2) Women expect equal pay regardless of merit; (3) Women possess greater legal rights and privileges; (4) Women are responsible for household chores; and (5) Women provoke men (and their self-victimization).

Nevertheless, the degree of attachment to each idea varies.

The concept with which participants show the least attachment is that women should be responsible for household chores (11.47%). Participants indicated skepticism toward this idea, as neither they nor their peers were perceived to agree with it. This perspective is perceived as antiquated and disconnected from contemporary youth (Baker, 2012). While movements such as #Tradwifes are emerging in some countries (Love, 2020), this discourse appears outdated and reminiscent of historical antifeminist rhetoric (Forrester, 2022). However, it is noteworthy that feminists were among the primary proponents of this discourse, possibly due to its historical significance in antifeminist discourse (Das Gupta, 2008).

This perception of household chores as an outdated and socially overcome issue may reflect the participants' immediate environment, where more egalitarian practices are normalized. Nevertheless, when examining broader societal data, a persistent gender gap becomes evident. According to Spain's National Statistics Institute [Instituto Nacional de Estadística (INE), 2023], women still dedicate twice as much time as men to household tasks, revealing that gendered expectations regarding domestic responsibilities remain deeply ingrained in everyday life. This contrast between personal perceptions and structural realities underscores the complexity of social change processes.

It is important to clarify that gender equality policies are not designed to grant “privileges” to women, but rather to address structural inequalities and systemic discrimination. Institutions such as the European Institute for Gender Equality (EIGE—European Institute for Gender Equality, 2023) emphasize that measures like positive action or gender quotas are corrective tools aimed at ensuring equal opportunities, particularly in fields where historical imbalances persist. Framing these initiatives as “unfair advantages” reflects a misinterpretation of their purpose and societal necessity.

Beyond this specific belief, a broader phenomenon emerges: the disconnection between the general support for gender equality and the reluctance to identify with feminism. This trend is not exclusive to our participants. For instance, the 2023 Youth and Gender Barometer (Fundación FAD Juventud, 2023) revealed that while 78% of young people in Spain support gender equality, only 42% self-identify as feminists. Our own findings mirror this pattern: participants reported strong support for equality values, yet their level of feminist self-identification was noticeably lower. This discrepancy illustrates how antifeminist narratives have successfully distorted the meaning of feminism, portraying it as an extremist or divisive ideology rather than as a movement advocating for human rights and social justice.

In the legal domain, the “Men's rights” movement focuses on two primary areas of concern. Firstly, it addresses allegations of a sexual nature, including discussions on false allegations and consent (Gotell and Dutton, 2016). This discourse gained significant traction in Spain during the enactment of the Integral Guarantee of Sexual Freedom Law, colloquially known as the “Only yes is yes” law in Spain in 2022. Critics of these movements accused the legislation of violating the presumption of innocence and equality before the law (Valdés, 2022).

The belief that false allegations of sexual violence are frequent remains a persistent narrative within antifeminist discourses. However, empirical studies consistently indicate that such cases represent a small minority of reported incidents. Research has shown that the prevalence of false reports typically ranges between 2% and 10% (Lisak et al., 2010; Rape Crisis Scotland, 2022). Specifically in Spain, the Public Prosecutor's Office (Fiscalía General del Estado, 2023) reported that false accusations in gender-based violence cases represented only 0.0096% of total complaints. This negligible percentage aligns with global estimates and directly challenges the widespread belief that false allegations are common. Persisting in this misconception contributes to a climate of suspicion and blaming toward victims, undermining both social and institutional efforts to combat gender-based violence (Abdelaziz, 2025).

Moreover, the results also hint at individuals advocating for “father's rights,” albeit with less prominence, likely influenced by the age distribution of the participants. These individuals typically comprise divorced or separated fathers from marital unions who voice grievances regarding legal impediments hindering their parental rights (Flood, 2012). Notably, certain factions within these father's rights groups espouse antifeminist sentiments, asserting their advocacy as a means to safeguard children who are purportedly “victims of feminist ideology” (Behre, 2015).

Slightly more prevalent is the notion that women are provocative since the participants themselves express disagreement, yet their friends and young people, in general, tend to endorse this idea to a moderate extent. This idea is clearly rooted in rape culture, a societal phenomenon where sexual assault and rape are normalized, trivialized, or excused (Johnson and Johnson, 2021). Victim-blaming, a significant aspect of rape culture, involves holding women accountable for any future aggressions they may suffer based on their behavior or, as observed in this case, their attire, rather than placing responsibility on their perpetrators (Stubbs-Richardson et al., 2018).

It should be noted that beyond the arguments described so far, the most recurrent idea was that feminism does not pursue equality. Despite participants' disagreement with this notion, they believe that it is endorsed by their friends and young people in general. Particularly striking is the prevalence of this belief among the youngest participants. Moreover, a significant disparity emerges between participants' perceptions of feminism and their stance on gender equality. While they strongly support gender equality (with a mean rating of 4.83 on a 1–5 scale), their self-identification as feminists falls almost one point lower (3.96). This discrepancy suggests a partial dissociation between the concepts of feminism and the pursuit of gender equality among the participants; these concepts are not perceived as entirely synonymous.

This idea is connected to the discrediting of the feminist movement by various political actors, former right-wing, ultra-conservative, religious groups, and the aforementioned Men's Rights Movement (Unal Abaday, 2021). Their claims resonate with the notion that “women have gone too far” by discriminating against them (Elder et al., 2021). They critique women and their advancements, arguing that they are stripping power away from men and thus frequently oppose certain female advancements and current equality initiatives (Farci and Righetti, 2019). Much of their focus is directed toward critiquing feminism, which they derogatorily label as “feminazism” or misrepresent as “radical feminism,” distorting its original theoretical foundations to portray it as an anti-male cultural threat (Bou-Franch and Blitvich, 2014). Organizations such as FREE or NCFM in the USA represent this current, with some of their leaders being former anti-sexists of the 1970s (Bonino, 2002). In Europe (Paternotte and Kuhar, 2018) and Spain, these ideas have gained traction in recent years, with media outlets criticizing and questioning equality plans (Bernardez-Rodal et al., 2022). Furthermore, it appears that these movements have influenced Spanish society, as evidenced by the aforementioned CIS survey, which indicates that 44.1% of men and 32.5% of women strongly or somewhat agree with this idea in Spain (CIS, 2024).

Finally, the idea that resonates most strongly with our participants is that women, or at least some of them, expect to receive equal pay for their work, even if they do not deserve it. It is worth noting that participants are moderately supportive of this notion, believing that both their friends and young people in general are largely in agreement. Within this discourse, they contend that it is untrue that women are currently paid less than men for the same work and cite several professions where men possess “greater skills” and thus should be compensated more (such as in sports like soccer or official capacities such as firefighters or police) (Eppard and Blau, 2020). However, empirical data refutes this perception. According to Eurostat (2023), the gender pay gap in Spain stands at 10.4%, even after adjusting for factors such as occupation, working hours, and experience. This indicates that structural inequalities, rather than differences in merit, persist in the labor market. Furthermore, the Global Gender Gap Report 2024 (World Economic Forum, 2024) highlights that Spain has made progress in reducing gender disparities but still ranks 17th globally in economic participation and opportunity for women. These findings underscore that the wage gap remains a tangible and documented issue, contrary to the participants' beliefs.

It is interesting to observe that soccer is repeatedly mentioned, as the sports industry in general, and soccer specifically, exhibits significant wage disparities (Wicker et al., 2023). Similarly, the other sector referenced, that of governmental bodies, remains heavily male-dominated (Perrott, 2016). In essence, both are fields where feminist advocacy still has considerable ground to cover, and this type of ideology represents a major stumbling block in these efforts for gender equality (Reid et al., 2018).

Finally, it is worth noting the differences in attachment they show, on average, with antifeminist ideas. While they perceive these ideas as quite distant from their own beliefs, they also perceive them as being further removed from their own beliefs compared to those of their friends or young people in general (Calder-Dawe and Gavey, 2016; Swirsky and Angelone, 2016). This pattern has also been observed in previous studies on feminism, where social desirability biases lead participants to respond in a way they feel is “politically correct,” projecting their thoughts onto the voices of others (Elder et al., 2021).

### 4.1 Practical implications and recommendations

The findings of this study reveal that antifeminist ideas are still present among young people pursuing education degrees, addressing a variety of issues that warrant attention. Despite being a population expected to be more sensitive to gender equality, these future educators show varying degrees of attachment to discourses that contradict feminist principles.

These results underscore the necessity for educational institutions to take an active role in debunking antifeminist myths, promoting critical thinking, and fostering feminist perspectives within teacher training programs. Developing a feminist identity is closely linked to the institutional environments that nurture and support such viewpoints (Aronson, 2003). Therefore, universities and educational programs should integrate feminist perspectives into their curricula, organize debate forums to address antifeminist discourses with empirical evidence, and establish gender equality committees aimed at promoting equity and diversity in educational settings.

Furthermore, addressing these misconceptions is not merely an academic challenge but aligns with the United Nations' Sustainable Development Goal 5 (Gender Equality). Future educators play a pivotal role in shaping egalitarian values and preventing the perpetuation of gender stereotypes in schools. Hence, targeted interventions at the university level are crucial to equip these students with the necessary tools to foster inclusive and gender-equal learning environments.

### 4.2 Limitations and future research

This study has several limitations that should be acknowledged. First, the study utilized a non-probabilistic sample and employed a cross-sectional design within a specific setting—the northern region of Spain. Consequently, the findings drawn from this study may not be generalizable to other societies or contexts. Additionally, the academic disciplines chosen by our participants displayed a noticeable gender imbalance, which could influence the generalizability of the results. Exploring students in fields with a more equitable male-to-female ratio could offer valuable insights into how contextual factors influence them.

Moreover, prior research has indicated that students in university education programs are more likely to identify with feminism than young people in other contexts (Fernandez et al., 2023). Therefore, future research should aim to explore these antifeminist discourses across diverse educational fields and sociocultural contexts, including longitudinal studies that assess how these perceptions evolve over time.

Finally, it would be beneficial to further investigate the mechanisms through which antifeminist narratives are socialized among young people, particularly in digital and peer environments. Understanding these dynamics is essential for developing effective educational strategies to counteract misinformation and foster a deeper, more critical engagement with gender equality and feminist perspectives.

In summary, throughout history, every wave of feminism has sparked antifeminist movements in response. Today, the recent feminist milestones achieved in the country are also giving rise to antifeminist discourses influencing the younger population. Therefore, it is essential to understand these discourses and establish platforms for open dialogue to challenge them and educate future professionals committed to equality and feminism.

## Data Availability

The raw data supporting the conclusions of this article will be made available by the authors, without undue reservation.
